# Prediction of microRNAs affecting mRNA expression during retinal development

**DOI:** 10.1186/1471-213X-10-1

**Published:** 2010-01-06

**Authors:** Amit Arora, Jasenka Guduric-Fuchs, Laura Harwood, Margaret Dellett, Tiziana Cogliati, David A Simpson

**Affiliations:** 1 Centre for Vision and Vascular Science, Queen's University Belfast, Ophthalmic Research Centre, Institute of Clinical Science, Royal Victoria Hospital, Belfast BT12 6BA, UK

## Abstract

**Background:**

MicroRNAs (miRNAs) are small RNA molecules (~22 nucleotides) which have been shown to play an important role both in development and in maintenance of adult tissue. Conditional inactivation of miRNAs in the eye causes loss of visual function and progressive retinal degeneration. In addition to inhibiting translation, miRNAs can mediate degradation of targeted mRNAs. We have previously shown that candidate miRNAs affecting transcript levels in a tissue can be deduced from mRNA microarray expression profiles. The purpose of this study was to predict miRNAs which affect mRNA levels in developing and adult retinal tissue and to confirm their expression.

**Results:**

Microarray expression data from ciliary epithelial retinal stem cells (CE-RSCs), developing and adult mouse retina were generated or downloaded from public repositories. Analysis of gene expression profiles detected the effects of multiple miRNAs in CE-RSCs and retina. The expression of 20 selected miRNAs was confirmed by RT-PCR and the cellular distribution of representative candidates analyzed by *in situ *hybridization. The expression levels of miRNAs correlated with the significance of their predicted effects upon mRNA expression. Highly expressed miRNAs included miR-124, miR-125a, miR-125b, miR-204 and miR-9. Over-expression of three miRNAs with significant predicted effects upon global mRNA levels resulted in a decrease in mRNA expression of five out of six individual predicted target genes assayed.

**Conclusions:**

This study has detected the effect of miRNAs upon mRNA expression in immature and adult retinal tissue and cells. The validity of these observations is supported by the experimental confirmation of candidate miRNA expression and the regulation of predicted target genes following miRNA over-expression. Identified miRNAs are likely to be important in retinal development and function. Misregulation of these miRNAs might contribute to retinal degeneration and disease. Conversely, manipulation of their expression could potentially be used as a therapeutic tool in the future.

## Background

miRNAs are small RNA molecules of 18-22 nucleotides in length which regulate gene expression. Precursor RNAs transcribed either as independent genes or within introns of coding genes are processed by the RNAseIII enzymes Drosha and Dicer to form the mature miRNAs [1]. The first miRNAs were discovered as a result of their role in the timing of adult cell fate determination in *Caenorhabditis elegans*. Both *let-7 *and *lin-4 *are temporally regulated and cause a decrease in expression of target proteins which control developmental stage-specific events [2-4]. The significance of miRNAs in developmental processes has now been shown in many plants and animals [5].

There is growing evidence that miRNAs play an important role in regulating eye development and function. In the mouse, prevention of miRNA maturation by conditional knockout in retinal tissue of the Dicer protein resulted in formation of photoreceptor rosettes and retinal degeneration and had a profound effect upon visual function, as measured by electroretinogram (ERG) [6]. Li and Carthew reported reciprocal negative regulation between Yan protein and miR-7 in *Drosophila *retinal cells: Yan directly represses the expression of miR-7 in undifferentiated progenitor cells and miR-7 directly represses the expression of Yan in differentiated photoreceptor cells [7]. The switch between these mutually exclusive expression states occurs when Yan degradation is triggered by epidermal growth factor receptor (EGFR) signaling.

Expression of various miRNAs has been demonstrated in the retina [8-10], notably a sensory organ-specific polycistronic cluster comprising miR-96, miR-182, and miR-183 [11]. The profile of miRNAs expressed in the retina has been shown to change during degeneration in several mouse models [12,13]. Many genes with retinal-specific functions are predicted to be targeted by miRNAs and functional interactions have been demonstrated [14].

miRNAs were initially thought to act through inhibition of protein translation [2]. Whilst this mechanism has been widely confirmed [15] and the global impact of miRNAs on protein levels demonstrated [16,17], it has become apparent that miRNAs also affect mRNA levels. This was first clearly demonstrated by the observation that those mRNAs with reduced expression following over-expression of a specific miRNA were enriched for predicted target sites of that miRNA [18]. Many miRNAs display tissue-specific expression [19] and inhibit expression of genes which are found at lower levels relative to other tissues [18,20]. The action of miRNAs upon mRNA levels has been widely confirmed in subsequent studies [21,22], which have also improved understanding of the properties of functional predicted target sites [16,23].

Inhibition of gene expression by miRNAs is mediated by base pairing between the miRNA and complementary sequences in the 3'UTR of target genes. However, unlike siRNAs, which direct cleavage of perfectly matched sequences, miRNAs have imperfect complementarity with their target sites. The features required for a functional target site are not fully understood, but include a stretch of perfect complementarity with a ~7 nucleotide 'seed' region at the 5' end of the miRNA [24]. A range of target prediction algorithms have been developed and each miRNA is thought to target approximately 200 transcripts [25,26]. Over 550 human miRNAs have been defined to date (miRBase v12 [27,28]) and have been suggested to regulate 30% of genes [24].

We have shown recently that the effects of miRNAs upon mRNA levels are such that the consequences of expression of a specific miRNA can be detected within a single gene expression profile [29]. Here this approach is applied to three experimental samples chosen to indicate candidate miRNAs affecting mRNA levels in ciliary epithelium-derived retinal stem cells from the adult eye (CE-RSCs), the developing retina during rod photoreceptor differentiation (postnatal murine retina) and in the mature retina (adult murine retina). This analysis may be indicative of those miRNAs with greatest effects upon target gene expression, or may reveal a subset with targets which are more susceptible to mRNA degradation. In either case, demonstration of the expression of these miRNAs confirms them as candidates for future manipulation aimed at directing differentiation or maintaining the mature retina.

## Results

### In silico prediction of candidate miRNAs affecting mRNA levels

**Figure 1:**
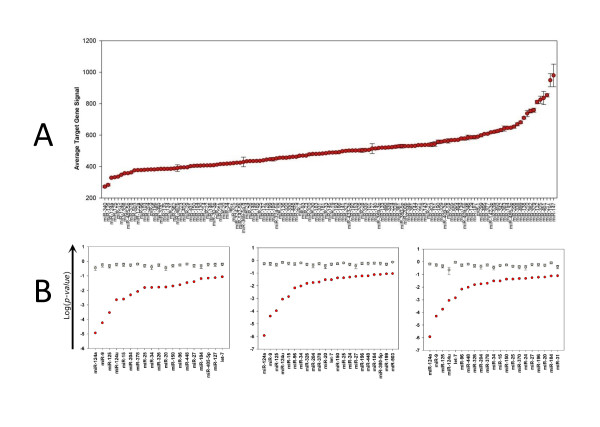
**miRNA target gene expression**. (A) miRNAs ranked by target gene expression levels (Average Target Gene Signal) in P4 retina. (B) Probabilities of each miRNA having a significant effect (p < 0.1) upon target gene expression according to Wilcoxon ranked sum test. The log_10_(p-value) for each miRNA (x-axis) is plotted as a red circle and the mean probability (± standard error) derived from five random sets of predicted target genes is plotted in grey.

Each miRNA was mapped to its predicted target genes in each mRNA expression profile and the average levels of the transcripts of their target genes were calculated. The ranked average target gene expression values in the P4 retina are shown in Figure 1A. The probability that each miRNA has a significant effect upon gene expression was calculated using the Wilcoxon ranked sum test; the results for P4 retina are illustrated in Figure 1B. The predictions from all gene expression datasets are listed in Additional file 1: Table S1 and the significant combined probabilities are shown in Table 1 (p < 0.05). Multiple mRNA expression profiles were analysed for murine CE-RSCs, P4 and adult retina. Significant predicted effects upon mRNA expression were detected for multiple miRNAs in the P4 and adult retina and for a smaller number in CE-RSCs. At P4 many miRNAs had highly significant effects, with miR-124, miR-125 and miR-9 being particularly significant (Figure 1, Table 1 and Additional file 1: Table S1). In the adult retina miR-124 and miR-125 were again prominent, but others, including miR-24, miR-326, miR-370, miR-96 and let-7 also had highly significant predicted effects. The greater number of genes assayed for the adult retina may have contributed to the detection of several more miRNAs with significant predicted effects and the smaller p-values than at P4. Whilst most of the candidate miRNAs predicted to affect mRNA levels (P < 0.05) at P4 were also in the adult, only miR-125, miR-378 and miR-24 were detected in all the different tissue types and developmental stages. Gene expression data from human CE-RSCs from adult eyes were also analysed and although many different miRNAs were predicted to be affecting mRNA levels, it was notable that the two miRNAs with the most significant effects in murine CE-RSCs, miR-378 and miR-485-3p, were also the 3^rd ^and 4^th ^most significant in human CE-RSCs (Additional file 1: Table S1).

**Table 1:** miRNAs predicted to affect retinal gene expression.

CE-RSCs		P4		Adult	
**miRNA**	**Probability**	**miRNA**	**Probability**	**miRNA**	**Probability**

miR-485-3p	2.25E-03	miR-124a	5.59E-11	miR-125	8.01E-18

miR-378	1.08E-02	miR-9	2.29E-10	miR-124a	2.86E-15

miR-30-3p	1.14E-02	miR-125	3.92E-09	let-7	8.96E-09

miR-125	1.30E-02	miR-15	1.44E-07	miR-24	6.94E-08

miR-153	2.14E-02	miR-124u	8.98E-05	miR-96	6.61E-07

miR-342	2.46E-02	miR-34	3.36E-04	miR-370	9.17E-07

miR-142-5p	2.59E-02	miR-204	3.37E-04	miR-326	1.68E-06

let-7	2.69E-02	miR-378	3.55E-04	miR-29	7.82E-06

miR-24	2.99E-02	miR-20	5.95E-04	miR-331	9.74E-06

miR-183	3.00E-02	miR-96	6.40E-04	miR-124u	5.14E-05

miR-370	4.42E-02	miR-25	1.05E-03	miR-20	8.02E-05

miR-33	4.43E-02	miR-27	1.65E-03	miR-27	5.56E-04

		let-7	2.20E-03	miR-196	1.32E-03

		miR-326	2.79E-03	miR-485-5p	2.87E-03

		miR-150	8.95E-03	miR-34	3.35E-03

		miR-196	1.26E-02	miR-378	4.73E-03

		miR-184	1.33E-02	miR-127	5.00E-03

		miR-448	1.56E-02	miR-30	6.74E-03

		miR-503	2.76E-02	miR-150	1.21E-02

		miR-485-5p	3.33E-02	miR-128	1.32E-02

		miR-30	3.44E-02	miR-15	2.30E-02

		miR-24	3.98E-02	miR-9	2.62E-02

		miR-29	4.29E-02	miR-214	2.69E-02

				miR-204	2.93E-02

				miR-184	4.05E-02

The miRNAs with significant effects (combined p < 0.05) upon mRNA expression in mouse adult CE-RSCs, P4 and adult retina.

**Figure 2:**
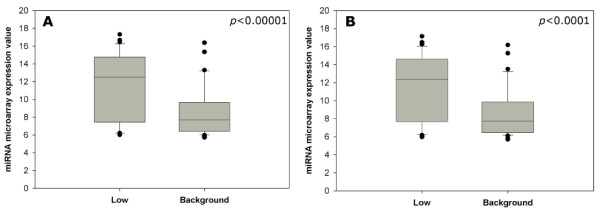
**Association of miRNA expression with extent of predicted effect upon target mRNA expression in adult murine retina**. Expression of those miRNAs with a significant effect (p < 0.1) upon target gene expression ('low' group) was significantly higher than that of those miRNAs with no predicted effects ('background' group). Microarray data from Loscher *et al *[13] was available for two strains of mouse, C57 (A) and 129 (B), and both showed a similar result.

We hypothesized that miRNAs with detectable effects upon mRNA expression are more highly expressed than those with no effect. We tested this hypothesis in the adult mouse retina using miRNA expression data published by Loscher *et al*. [13]. A list was compiled of miRNAs whose predicted target genes exhibited significantly low expression. Expression of miRNAs within this group was significantly higher than that of those miRNAs for which no effect on target mRNAs was predicted (Figure 2).

### Experimental analysis of miRNA expression

The following miRNAs had highly significant predicted effects on target mRNA levels and were selected for analysis by RT-PCR: miR-124, miR-125, miR-9, and miR-24. A range of other candidates with p-values < 0.05 in at least one experimental condition (miR-128, miR-150, miR-204, miR-25, miR-27, miR-326 miR-34, miR-370, miR-378 and miR-485-5p) were selected to represent different predicted patterns of activity or for their lack of previous association with neural tissue. In addition, miR-122, which had p < 0.1 in CE-RSCs and P4 and adult retina, was of interest because it has been previously characterised as a highly expressed, liver-specific miRNA [19,22,30]; the possibility of an alternative role in the retina is intriguing. Let7d has previously been shown to be highly expressed in the retina [14] and was used as a positive control.

**Figure 3:**
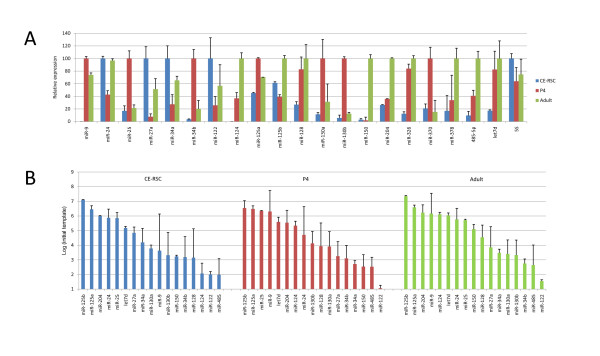
**miRNA expression detected by RT-PCR**. (A) Relative quantification of selected miRNAs in porcine CE-RSCs and murine P4 and adult retina. (B) Absolute miRNA expression levels estimated from qRT-PCR data.

**Figure 4:**
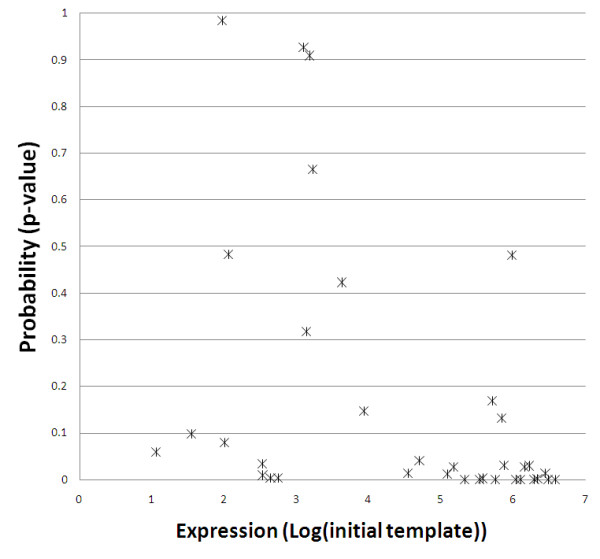
**Comparision between the predicted effects of miRNAs and their expression as determined by RT-PCR**. The estimated copy number (Log(initial template)) of each miRNA in a given sample is plotted against the probability (Wilcoxon ranked sum test) that it is having an effect upon target gene expression. The most highly expressed miRNAs have very significant effects upon predicted target gene expression and the overall correlation between miRNA expression and significance of effect upon target gene expression is significant (p = 4.0E-04; Spearman rank correlation).

The pattern of relative expression in the three samples considered (porcine CE-RSCs, mouse P4 and adult retina) varied widely amongst miRNAs (Figure 3A). The expression of each individual miRNA corresponded broadly with its predicted effects upon target gene expression (e.g. miR-25 highest at P4 and miR-124 absent in CE-RSCs but present in P4 and adult mouse retina). However, whilst this analysis indicates the sample in which the relative expression of a single miRNA is highest, it gives no indication of the inter-miRNA variation in expression in that sample. It is also important to consider the relative absolute expression of different miRNAs within a single sample when considering the impact of each upon target mRNA expression. Therefore, starting template copy numbers were estimated for each miRNA based on threshold cycle (Ct) and amplification efficiency or by the linear regression efficiency method [31], which were broadly in agreement (Additional file 2: Figure S1). The miRNAs measured in each sample are shown in Figure 3B ranked by their starting template copy number in the RT-PCR assays. Those miRNAs with the most significant predicted effects upon mRNA expression are highly expressed. Notably, miR-125 has amongst the most significant predicted effects upon mRNA expression at all stages (Table 1) and miR-125a and miR-125b are the most highly expressed miRNAs in all samples (Figure 3B). Furthermore, miR-124 is highly expressed in P4 and adult retina in accordance with its predicted effects. To test the expected relationship between predicted effects and miRNA expression, the estimated miRNA copy numbers were plotted against predicted probability (Figure 4). This revealed a significant correlation (Spearman rank correlation, p = 4.0E-04), with the majority of most highly expressed miRNAs having highly significant predicted effects.

**Figure 5:**
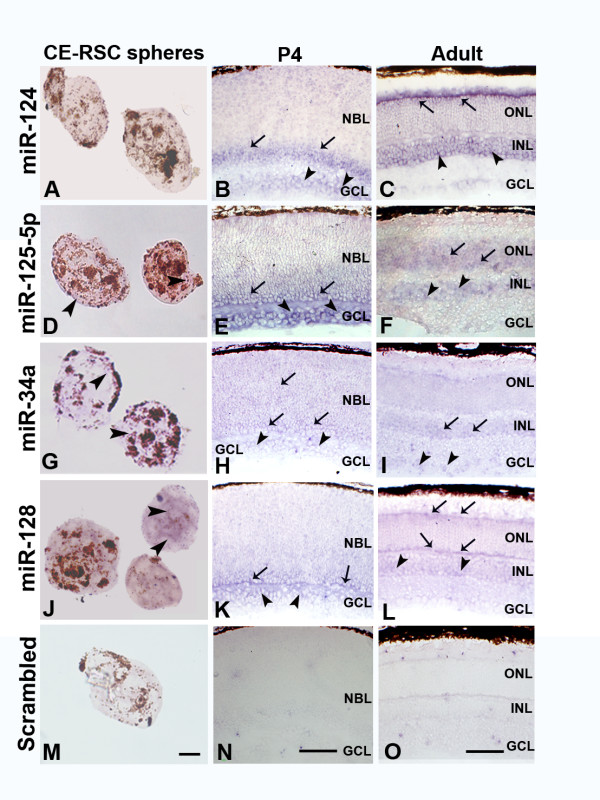
**Cellular expression of selected miRNAs**. ISH was performed on porcine CE-RSC neurospheres, and P4 and adult mouse retinas. Some neurosphere cells are naturally pigmented (dark brown). **A**: No positive signal was detected for miR-124 in CE-RSC neurospheres. **B**: Expression of miR-124 at P4 corresponded to the location of amacrine (arrows) and ganglion cells (arrowheads). **C**: In the adult retina, miR-124 was expressed in all layers with the highest intensity in the photoreceptor inner segments (arrows) and cells in the INL (arrowheads). **D**: miR-125b-5p was detected in the RSC neurospheres (purple staining depicted with arrowheads). **E**: In the P4 retina strong expression of miR-125b-5p was found in the inner portion of the NBL (arrows) and GCL (arrowheads). **F**: In the adult retina expression of miR-125b was detected in the ONL (arrows) and INL (arrowheads). G: Weak hybridisation signal for miR-34a was observed in CE-RSC neurosphere cells (arrowheads). H: P4 retina displayed week miR-34a signal throughout NBL (arrows) and ganglion (arrowheads) **I**: In the adult retina the strongest signal for miR-34a was observed in the INL (arrows) and in some cells in the GCL (arrowheads). **J**: Strong hybridisation signal was detected for miR-128 in the CE-RSC neurospheres (arrowheads). **K**: In the P4 retina a hybridisation signal for miR-128 was observed in the inner portion of the NBL (arrows) and in some cells of the GCL (arrowheads). **L**: In the adult retina strongest miR-128 hybridisation signal was detected in the INL (arrowheads), the outer portion of the outer plexiform layer and photoreceptor inner segments (arrows). ISH with scrambled negative control gave no signal in CE-RSCs (M), P4 (N), or adult mouse retina (O). NBL-neuroblast layer, GCL-ganglion cell layer, INL-inner nuclear layer, ONL-outer nuclear layer. Scale bars 50 μm.

*In situ *hybridization (ISH) was performed to localise expression of selected miRNAs (Figure 5). Expression in the adult murine retina of the well-characterised neural miRNA miR-124 concurred with published reports [9]. In P4 retina, miR-124 positive signal was mainly colocalised with ganglion and amacrine cells. Expression of miR-34a, which has not previously been described in the retina, was detected in the porcine CE-RSC neurospheres and throughout the mouse P4 retina. In the adult mouse retina the strongest signal was observed in the inner nuclear layer (INL) and in some cells in the ganglion cell layer (GCL). miR-128 was expressed in all samples, notably in CE-RSC neurospheres and the inner plexiform layer of the retina at P4. miR-125b was also expressed at all stages with strong signal in the inner plexiform layer at P4. In the adult retina miR-125b was expressed in the inner and outer nuclear layers. No expression was observed for miR-122 and miR-378 (data not shown).

### Predicted targets of candidate miRNAs affecting mRNA levels

**Figure 6:**
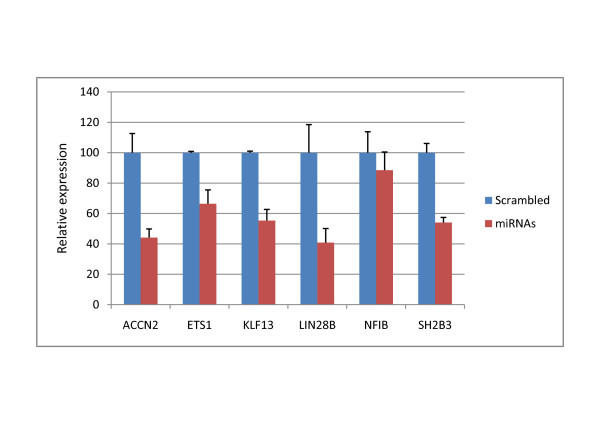
**Overexpression of miRNAs reduces predicted target mRNA levels**. Pools of miR-124, miR-125 and miR-9 miRNA mimics (miRNAs) or scrambled controls (Scrambled) were transfected into HEK293 cells. The mRNA expression of six genes predicted to be targeted by these three miRNAs was subsequently assessed by qRT-PCR. The graph shows the significant (P < 0.01) reduction in expression of ACCN2, ETS1, KLF13, LIN28B and SH2B3 following transfection with miRNAs relative to the scrambled control (error bars show standard deviation). There was no significant difference in expression of NFIB.

In order to identify the genes most likely regulated by miRNAs in CE-RSCs, P4 and adult retina, the target predictions for all the miRNAs shown to have a significant effect upon gene expression were combined. This revealed that some genes were predicted to be targeted by almost half of the miRNAs identified in each sample (Table 2 and Additional file 3: Table S2). These genes are the most likely to be real miRNA targets because if some predicted sites are not functional others may be active. Also, the co-operative action of multiple miRNAs may result in greater effects [23]. Six genes targeted by three miRNAs with the highest expression and greatest predicted effects (miR-124; miR-125 and miR-9.) were selected for validation: ACCN2; ETS1; KLF13; LIN28B; NFIB and SH2B3. Following transfection of HEK293 cells with a pool of miR-124, miR-125 and miR-9 miRNA mimics the mRNA expression of 5 of these 6 genes was significantly reduced (Figure 6).

**Table 2:** Genes predicted to be targets of the candidate miRNAs affecting mRNA levels.

CE-RSCs		P4		Adult	
**Gene**	**miRNAs**	**Gene**	**miRNAs**	**Gene**	**miRNAs**

CUGBP2	153; 24; 183; 378; 33; 30-3p; 342	ACVR2A	27; 96; 29; 128; 196; 150; let-7/98; 124.1; 125/351; 362; 378	ACVR2A	503; 27; 96; 448; 378; 29; 196; 125/351; 150; 124.1; let-7/98

KPNA4	142-5p; 33; 153; 378; let-7/98; 183; 24	TNRC6B	362; 184; 27; 124.1; 9; 204/211; 378; 485-5p; 150; 29; 22	TNRC6B	150; 503; 378; 448; 9; 184; 485-5p; 29; 204/211; 124.1; 27

ZNF148	183; 125/351; 370; 142-5p; 24; 378; 153	**NFIB**	125/351; 331; 22; 128; 485-5p; 124.1; 29; 27; 9; 24	RICTOR	196; 448; 124.1; 96; 503; 326; 27; let-7/98; 204/211; 29

CLCN5	30-3p; let-7/98; 378; 24; 142-5p; 153	ZNF148	34b; 196; 204/211; 378; 124.1; 24; 128; 370; 125/351; 27	CLCN5	448; 503; 378; 9; 29; 24; let-7/98; 27; 485-5p

CPEB4	142-5p; 33; 342; 125/351; 153; let-7/98	MECP2	34b; 22; 204/211; 378; let-7/98; 196; 326; 96; 124.1	HMGA2	196; 125/351; 204/211; 9; 485-5p; 503; 150; 326; let-7/98

DAGLA	153; 183; 485-3p; 125/351; 24; let-7/98	RP11-130N24.1	362; 96; 9; 29; 125/351; 196; 27; 34b; 124.1	ZNF148	204/211; 196; 124.1; 34b; 125/351; 24; 448; 27; 378

EPHA4	153; 125/351; 33; 30-3p; let-7/98; 183	SP1	125/351; 29; 24; 27; 326; 378; 22; 128; 124.1	CPEB3	196; 125/351; 503; 29; let-7/98; 9; 27; 34b

NARG1	153; 378; 33; 342; 30-3p; 370	ZBTB39	9; 128; 485-5p; 22; 124.1; let-7/98; 27; 331; 370	MECP2	326; 34b; 378; let-7/98; 96; 196; 124.1; 204/211

PTPRD	485-3p; let-7/98; 30-3p; 153; 24; 142-5p	ATXN1	125/351; 29; 9; 370; 326; 96; let-7/98; 34b	**NFIB**	124.1; 448; 485-5p; 29; 9; 24; 27; 125/351

ACVR2A	125/351; 153; 378; let-7/98; 183	CCNJ	let-7/98; 196; 27; 128; 204/211; 370; 125/351; 96	RP11-130N24.1	9; 125/351; 124.1; 27; 29; 196; 96; 34b

BTBD7	342; 30-3p; 153; 378; 142-5p	CLCN5	29; 9; 485-5p; 378; 24; 128; 27; let-7/98	AKT3	124.1; 150; 503; 29; 448; 326; 125/351

CPEB2	153; 33; let-7/98; 183; 142-5p	CPD	196; 204/211; 370; 128; 150; let-7/98; 331; 27	ATXN1	34b; 125/351; 326; 96; let-7/98; 29; 9

DDX3X	30-3p; 33; 370; 183; 342	CPEB3	128; 9; let-7/98; 125/351; 196; 29; 27; 34b	BRPF3	96; 326; 204/211; 125/351; 27; 503; 9

E2F3	125/351; 153; 370; 378; 342	HMGA2	196; 150; 485-5p; 204/211; 326; 125/351; 9; let-7/98	BSN	448; 124.1; 24; 125/351; 150; 9; 503

FBXO33	; 142-5p; 30-3p; 342; 33; 153	IGF2BP1	370; 331; let-7/98; 196; 24; 96; 326; 9	CCND2	29; 196; 503; 96; let-7/98; 448; 124.1

MLL2	; 142-5p; 342; let-7/98; 370; 33	QKI	128; 485-5p; 9; 125/351; 370; 24; 124.1; 27	CCNJ	204/211; 27; 503; 196; 96; 125/351; let-7/98

PDE4D	; 183; 33; 24; 378; 485-3p	RICTOR	27; 196; 96; 204/211; 29; 326; 124.1; let-7/98	**KLF13**	9; 96; 125/351; 29; 448; 124.1; 378

QKI	; 24; 370; 33; 125/351; 142-5p	RIMS4	9; 96; 22; 27; 362; 127; 326; 331	MGA	326; 124.1; 378; 29; 9; let-7/98; 485-5p

RNF165	; let-7/98; 153; 24; 33; 142-5p	STC1	485-5p; 124.1; 9; 125/351; 22; 378; 34b; 96	ONECUT2	27; 29; let-7/98; 485-5p; 9; 96; 503

RSBN1	; 342; 183; 153; 378; 485-3p	BRPF3	27; 9; 96; 128; 125/351; 204/211; 326	PAPPA	27; 96; 150; let-7/98; 503; 448; 326

SETD7	; 485-3p; 153; 33; 125/351; 342	CPEB4	362; 96; 125/351; 128; 9; 27; let-7/98	PTPRD	24; 124.1; 204/211; 29; 503; let-7/98; 448

SIX4	; 153; 378; 33; 30-3p; 142-5p	DCX	27; let-7/98; 128; 29; 362; 9; 96	QKI	125/351; 124.1; 9; 503; 24; 485-5p; 27

SLC4A4	; 153; 125/351; 370; 142-5p; let-7/98	DTNA	9; 124.1; 378; 22; 24; 27; 128	RNF165	124.1; 448; let-7/98; 485-5p; 150; 29; 24

SOX11	; 125/351; 153; 33; 142-5p; 485-3p	FRMD4A	29; 9; 204/211; 128; 124.1; 96; 34b	SP1	326; 124.1; 125/351; 29; 378; 27; 24

SP1	; 24; 125/351; 485-3p; 378; 33	GABBR2	128; 9; 22; 378; 326; 204/211; 370	STC1	485-5p; 378; 34b; 124.1; 9; 125/351; 96

STC1	; 125/351; 30-3p; 142-5p; 378; 183	ISL1	27; 29; 128; 96; 9; 362; 378	ZFHX4	204/211; 96; 503; 485-5p; 448; 27; 9

THRAP1	; 485-3p; 142-5p; 183; 153; 24	KLF12	34b; 370; 204/211; 124.1; 29; 9; 27	**ACCN2**	9; 29; 326; 125/351; 124.1; 27

YOD1	; let-7/98; 183; 24; 30-3p; 125/351	MGA	378; 485-5p; 326; 29; let-7/98; 9; 124.1	APPBP2	9; 448; 378; 27; let-7/98; 29

ZFPM2	; 183; 142-5p; 485-3p; 153; 33	MITF	27; 331; 34b; 378; 124.1; 485-5p; 96	CPEB4	448; 9; 27; 125/351; 96; let-7/98

ARID4B	; 370; 183; 378; 142-5p	MMP16	150; 27; 370; 24; 124.1; 9; 96	CUGBP2	96; 326; 34b; 378; 196; 24

The top 30 genes in each tissue ranked by number of different miRNAs predicted to target them. Genes for which certain miRNA interactions were assessed experimentally are marked in bold. The full list of genes targeted by at least one of the candidate miRNAs affecting mRNA levels is shown in Table S2.

Comparison of the predicted miRNA target genes with those known to be involved in retinal function and disease [14] highlighted potential genes of interest (Table 3 and Additional file 3: Table S2). There was considerable overlap between known 'retinal genes' targeted by multiple miRNAs in P4 and adult retina. For example, a gene involved in the elongation of very long chain fatty acids (ELOVL4) is a predicted target of miRNAs with the most significant predicted effects on gene expression and the highest expression in RT-PCR assays. ELOVL4 has been implicated in Stargardts disease and macular dystrophy [32] and is expressed in photoreceptor inner segments [33], in agreement with the ISH localisation of miR-124 (Figure 5), its predicted regulator. Likewise, Neurocalcin delta (NCALD) is expressed in retinal amacrine and ganglion cells [34] in agreement with the expression of miR-204, one of its potential regulators [9].

**Table 3:** Predicted target genes known to be involved in retinal function and disease.

CE-RSCs		P4		Adult	
4: NLK	1: NEFL	6: CAMTA1	1: COL18A1	5: NCOR2	1: DDIT4

3: DMD	1: PCDHGC3	6: NCOR2	1: COL2A1	4: CAMTA1	1: DUSP1

2: ADD1	1: PPP1R3F	5: ELOVL4	1: DUSP1	4: ELOVL4	1: FAM57B

2: CAMTA1	1: PSAP	5: NCALD	1: FAM57B	4: NCALD	1: H2AFZ

2: GNB1	1: RARB	4: NLK	1: H2AFZ	4: NLK	1: HSP90AB1

2: GPX3	1: RARG	4: RARG	1: IGFBP5	4: TP53INP2	1: IGFBP5

2: GRIA4	1: RDH10	4: TSPAN9	1: IMPDH1	4: TSPAN9	1: IMPDH1

2: HSPA8	1: RGS16	3: FZD4	1: KIAA0143	3: DMD	1: KIF1B

2: JAG1	1: RHO	3: TEAD1	1: KIF1B	3: FZD4	1: MAK

2: PAX2	1: RIMS1	3: TP53INP2	1: MAK	3: JAG1	1: MAPT

2: RB1	1: SGIP1	3: ZNF385	1: MAPT	3: RARG	1: PCDHGC3

2: SLC17A7	1: SLC12A5	2: CFL1	1: PAX2	3: TEAD1	1: PITPNA

2: TEAD1	1: SLC25A18	2: CSDA	1: PITPNA	2: CSDA	1: PPP1R3F

1: APP	1: SPP1	2: DMD	1: PPP1R3F	2: GPX3	1: PRELP

1: ATP8B1	1: STAC2	2: GPX3	1: PRELP	2: GRIA4	1: PSAP

1: BHLHB3	1: SYP	2: GRIA4	1: PSAP	2: PAX2	1: RARA

1: CAMSAP1	1: TFAP2B	2: JAG1	1: RARB	2: SLC12A5	1: RARB

1: CCNI	1: TP53INP2	2: RARA	1: RB1	2: SLC17A7	1: RB1

1: CFL1	1: TSPAN9	2: RIMS1	1: RDH10	2: TRPM3	1: RDH10

1: CLASP2	1: USH2A	2: SGIP1	1: RGS16	2: ZNF385	1: RGS16

1: COL2A1	1: ZNF385	2: SLC12A5	1: RGS9	1: ACTB	1: RGS9

1: CP		2: SLC17A7	1: RHO	1: ACTG1	1: RHO

1: DDIT4		2: TAGLN	1: RS1	1: ADD1	1: RIMS1

1: DUSP1		2: TRPM3	1: SLC1A3	1: AFF1	1: RS1

1: EEF1A1		1: ACTB	1: SLC25A18	1: ANXA2	1: SGIP1

1: EIF1		1: ACTG1	1: SMOC2	1: ATP8B1	1: SLC25A18

1: ELOVL4		1: AFF1	1: SPARC	1: ATXN7	1: SMOC2

1: FZD4		1: ANXA2	1: SPG20	1: CAMK2D	1: SPARC

1: HSP90AB1		1: ATP8B1	1: STAC2	1: CCNI	1: SPG20

1: IGFBP5		1: ATXN7	1: SYP	1: CFL1	1: STAC2

1: KIF1B		1: CAMK2D	1: TFAP2B	1: CLASP2	1: SYP

1: MSH6		1: CCNI		1: COL11A1	1: TAGLN

1: NCALD		1: CLASP2		1: COL18A1	1: TFAP2B

1: NCOR2		1: COL11A1		1: COL2A1	

Genes predicted to be targeted by one or more candidate miRNA affecting mRNA levels were compared with genes implicated in retinal function[14]. The overlapping genes are listed, with the number of different miRNAs predicted to target them followed by the gene symbol.

To establish the cellular functions which are regulated by the miRNAs shown to affect mRNA expression, enrichment of functional annotation terms within the predicted target genes was assessed [35]. The genes with target sites for those miRNAs identified by our analyses were selected from the set of all genes with a conserved miRNA target site predicted by TargetScan; this list was therefore used as the background to assess enrichment (various categories, including many regulatory functions, are already enriched in this group in comparison to the whole genome; data not shown). In all the different tissue types at different developmental stages, genes with two or more predicted sites for candidate miRNAs affecting mRNA levels were enriched for annotations predominately relating to regulatory functions, notably gene transcription (Additional file 4: Table S3).

## Discussion

### miRNAs regulate mRNA expression in the retina

Gene expression is regulated by many factors at both transcriptional and posttranscriptional levels. The effect of miRNAs within these influences is detectable through analysis of microarray expression profiles; groups of mRNAs defined by presence of a putative target site for specific miRNAs were expressed at a significantly lower level than the group of non-target genes. These effects were observed in developing and adult retina and to a lesser extent in CE-RSCs. Not surprisingly, these effects were related to the expression level of the miRNA; those with extremely significant effects, such as miR-125, miR-124 and miR-9 were amongst the most highly expressed in the P4 and adult murine retina. The miRNA expression patterns determined in this study were consistent with previous reports. For example, miR-125, miR-124 and miR-9 have all been independently reported to be highly expressed in the retina [9-11]. The association between the expression level of a miRNA and its predicted effects on mRNA expression is robust because it was demonstrated for both our qRT-PCR analysis of a relatively small number of miRNAs and for global miRNA expression profiles determined by other investigators [13].

Genes predicted to be targeted by multiple candidate miRNAs affecting mRNA levels are the best candidates for *in vivo *regulation by this mechanism. It is difficult to test this assertion in retinal cells, however the predicted effects on mRNA expression were observed for five of six genes in an *in vitro *model. This provides some support to the predictions of individual genes affected by miRNAs, at least for those targeted by multiple miRNAs with highly significant overall effects on mRNA expression,

### The pattern of miRNA activity suggests a role in retinal differentiation

The predicted effects of miRNAs were less significant in mouse CE-RSCs than in the P4 and adult retina. miRNA expression was correspondingly lower, with only 4 of 20 miRNAs displaying highest expression in the CE-RSCs (although this may have been partly due to the porcine origin of the CE-RSCs used for RT-PCR analyses). The absolute levels of these miRNAs were also lower than those highly expressed in P4 and adult retina. This is consistent with reports that miRNA expression increases from very low levels at embryonic day 10, when the retina is comprised of retinal progenitor cells with similar properties and gene expression patterns to CE-RSCs [36], to higher levels in the P1 and adult retina [11]. This pattern of miRNA expression and predicted effects supports a role for miRNAs in retinal differentiation, maturation and maintenance of the adult retina. A similar observation was noted by Wienholds and co-authors, when they were able to detect only a few miRNAs during early zebrafish development [37].

Although generally lower than in retina, miRNA expression was described for the first time in this study in CE-RSCs. Human and porcine eyes are similar in terms of morphology and physiology. We have previously characterised porcine CE-RSCs [38] and therefore, although the availability of mouse expression data for CE-RSCs, P4 and adult retina made it the choice for in silico analyses, porcine CE-RSCs were used to confirm expression of predicted miRNAs. CE-RSCs from various species have been shown to have very similar properties and gene expression patterns [38-41] Of the 4 miRNAs expressed most highly in CE-RSCs according to RT-PCR, miR-24 was also predicted to have a significant effect (p < 0.05) upon mRNA expression and miR-122 had p = 0.08. It has recently been shown that miR-24a represses apoptosis and is required for proper retinal development [42]. These miRNAs could also play a role in maintaining the progenitor cell state, as has been shown in other tissues; over-expression of miR-24 causes a delay in maturation of hematopoietic progenitor cells [43] and over-expression of miR-122 delays differentiation of human embryonic stem cells [44].

Many of the specific miRNAs shown to affect mRNA expression in the P4 and adult retina have previously been implicated in neuronal differentiation. For example, miR-124 has been associated with the transition from neural progenitor to differentiated neuron in the zebrafish brain [45]. In mouse brain miR-124 expression is restricted to mature neurons [46] and it promotes neuronal differentiation by triggering brain-specific alternative pre-mRNA splicing [47]. The reported absence of miR-124 in neural and retinal stem/progenitor cells *in vivo *[9,48,49] concurs with the lack of detectable expression in the CE-RSC spheres. Expression of miR-124 in the immature retina (P4) was lower than that in the adult retina and the ISH signal at P4 mainly corresponded to early differentiating neurons, retinal ganglion and amacrine cells. Our ISH for miR-124 in the adult retina confirmed previous reports [9] and showed miR-124 expression in all retinal layers, with particularly high levels in photoreceptor inner segments.

miR-9 promotes progression of neurogenesis in the zebrafish brain [50]. Expression of miR-9 was not detected in CE-RSCs, but in contrast to miR-124 it peaked in the P4 retina. This pattern is in agreement with Xu *et al *[11], who reported a very low level of miR-9 expression at E10 which gradually increased and peaked at P10, being lower in the adult retina. Notably, the transcription factor Hes1, which maintains retinal progenitor pools during development is a predicted target for miR-9 [50]. Similarly to retinal progenitor cells, high levels of Hes1 mRNA are found in CE-RSC spheres (Yanagi et al 2006, our unpublished data). Decrease of miR-9 expression past P10 correlates with the completion of retinal cell differentiation. miR-125 is another miRNA with highly significant predicted effects upon mRNA expression and the high expression levels for miR-125a and miR-125b detected by RT-PCR are in agreement with previous miRNA array data [8,11]. Together with miR-124 and miR-9, miR-125b is also induced during neural differentiation of embryonic stem cells [51].

### miRNAs differ in their effects upon mRNA turnover and translational repression

Our results suggest that, although many miRNAs are present in the retina, only a few affect mRNA levels sufficiently to have a detectable influence on target gene expression. Despite conclusive evidence of the high expression of miRNAs such as miR-181, -182 and -183 in the retina [8,9] the genes targeted by these miRNAs did not show significant down regulation at the mRNA level in our analysis. Conversely miR-122, which has previously been shown to direct mRNA cleavage [21], was expressed at a low level, but had a significant predicted effect upon target gene expression in several samples (combined p = 0.08). Interestingly, miR-125b, one of the miRNAs with the strongest predicted effect upon mRNA levels, has been shown to accelerate deadenylation leading to rapid mRNA decay [52]. It is now accepted that miRNAs can mediate both translational repression and accelerated mRNA turnover [53]. One explanation for the detection of the effect of some highly expressed retinal miRNAs upon mRNA levels and not others could be that they are acting preferentially at the level of mRNA turnover.

## Conclusions

The evidence presented in this study suggests that many miRNAs affect the expression levels of their target mRNAs during retinal development. The enrichment of the predicted target genes of these miRNAs for regulatory functions is in keeping with the proposed regulatory role of miRNAs. The lack of a mRNA 'signature' for other miRNAs which are also known to be highly expressed in the retina, suggests that certain miRNAs are less active at the mRNA level and perhaps act predominately upon translation. The identity and expression pattern of those miRNAs which were detected by analysis of target gene expression, such as miR-124, miR-125 and miR-9, provides further evidence that miRNAs play a central role in neuronal differentiation during retinal development.

## Methods

### Animal procedures

All animal procedures were performed in compliance with the UK Animals (Scientific Procedures) Act 1986. Adult mice were euthanised in a CO_2 _chamber and P4 neonatal mice were lethally anesthetised by intra-peritoneal (IP) injection of an overdose of pentobarbitone. Eyes were enucleated and either processed for ISH or the retinas dissected and snap frozen in liquid nitrogen for RNA isolation.

### Isolation and culture of CE-RSCs

Mixed sex White Landrace pigs were obtained from the Department of Agriculture and Rural Development Northern Ireland, Hillsborough, UK. CE-RSCs were isolated and grown as described previously [38]. Briefly, 1-2 week old pigs were lethally anesthetised, eyes were enucleated and placed into oxygenated artificial cerebral spinal fluid [aCSF: 124 mM NaCl,5 mM KCl,1.3 mM MgCl2, 26 mM NaHCO3, and 10 mM D-glucose,(Sigma-Aldrich, Poole, UK)]. A strip of ciliary body was dissected and enzymatically digested in Hanks' Balanced Salt Solution (HBSS) containing 2 mg/ml dispase (Sigma-Aldrich, Poole, UK) for 20 minutes at 37°C, followed by digestion in EBSS containing 1.33 mg/ml trypsin, 0.67 mg/ml hyaluronidase and 78 units/ml collagenase (Sigma-Aldrich, Poole, UK) for 20 min at 37°C. The supernatant was decanted and replaced with serum-free medium (SFM, DMEM/F12 (1:1) containing 0.6% (w/v) glucose, 2 mM glutamine, 5 mM HEPES buffer, 2% (v/v) B27, 100 units/ml penicillin and 100 units/ml streptomycin) with 1 mg/ml trypsin inhibitor (Invitrogen, Paisley UK). The cellular debris was gently triturated and dissociated into single cells with a fire-polished pipette. Cells were pelleted at 800 rpm for 10 minutes, resuspended in SFM and passed through a 40 μm cell strainer (BD Biosciences, USA). Cells were counted and plated at a density of 3 × 10^4 ^cells/ml in SFM supplemented with 20 ng/ml of epidermal growth factor (EGF) and 10 ng/ml basic fibroblast growth factor (bFGF) (Invitrogen, Paisley, UK). After 7 days newly formed sphere colonies were collected, pelleted at 800 rpm for 10 minutes and digested in Accumax cell counting solution (ICT, San Diego, USA) for 20 min at RT and mechanically dissociated into single cells by pipetting. Cells were washed once in SFM and plated at a density of 3 × 10^4 ^cells/ml. After 7 days secondary spheres were collected.

### miRNA target confirmation

HEK293-T cells were transfected at 80% confluency with miRNA mimics (Qiagen, Crawley, UK) using Turbofect transfection reagent (Fermentas, York, UK). Cells were collected for RNA isolation at 48 hours post transfection.

### RNA isolation

For mRNA analyses RNA was extracted using an RNeasy Micro Kit with on column DNAse digestion (Qiagen, West Sussex, UK) according to the manufacturer's protocol. For miRNA analyses total RNA was extracted using a mirVana extraction kit (Ambion, Foster City, CA) according to the manufacturer's protocol.

### mRNA microarray analysis and gene expression datasets

RNA samples from retinas of three P4 mice were labeled and hybridized to GeneChip^® ^430A 2.0 mouse microarrays (Affymetrix, Santa Clara, Ca.) according to the manufacturer's protocols. Raw data were analysed using the Affymetrix MAS 5.0 algorithm and the results deposited in NCBI's Gene Expression Omnibus (GEO) [54,55]. Publicly available mRNA expression data were downloaded from GEO. All the data were generated from Affymetrix genechips. Samples analysed on the MOE430A platform were available for CE-RSC and are directly comparable with the Mouse 430A 2.0 platform used for the P4 retina samples (~14,000 well characterised transcripts). Data from the MOE430B platform (~22,000 transcripts) was also available for several CE-RSC samples. The Mouse430_2 platform used for adult retinal expression interrogated all the transcripts in the A and B platforms. The tissues and GEO sample accession numbers are listed in Table 4.

**Table 4:** Gene expression datasets.

GEO accession	Type	Organism	Tissue
**GSM87191**	mRNA	Human	Retinal spheres from ciliary epithelium of adult eye

**GSM87193**	mRNA	Human	Retinal spheres from ciliary epithelium of adult eye

**GSM87194**	mRNA	Human	Retinal spheres from ciliary epithelium of adult eye

**GSM87195**	mRNA	Mouse	Retinal spheres from ciliary epithelium of adult eye

**GSM87196**	mRNA	Mouse	Retinal spheres from ciliary epithelium of adult eye

**GSM87197**	mRNA	Mouse	Retinal spheres from ciliary epithelium of adult eye

**GSM87198**	mRNA	Mouse	Retinal spheres from ciliary epithelium of adult eye

**GSM87199**	mRNA	Human	Retinal spheres from ciliary epithelium of adult eye

**GSM87200**	mRNA	Human	Retinal spheres from ciliary epithelium of adult eye

**GSM87201**	mRNA	Mouse	Retinal spheres from ciliary epithelium of adult eye

**GSM87202**	mRNA	Mouse	Retinal spheres from ciliary epithelium of adult eye

**GSM32153 **[64]	mRNA	Mouse	Adult Retina

**GSM81691 **[65]	mRNA	Mouse	Adult Retina

**GSM92629 **[66]	mRNA	Mouse	Adult Retina

**GSM107514**	mRNA	Mouse	Adult Retina

GSM419995*	mRNA	Mouse	P4 Developmental Retina

GSM419996*	mRNA	Mouse	P4 Developmental Retina

GSM419997*	mRNA	Mouse	P4 Developmental Retina

The mRNA expression datasets analysed to detect the effect of miRNAs were downloaded from Gene expression omnibus (GEO) or created in-house (*). The GEO accession numbers and details are listed. Numbers indicate associated references.

### miRNA target predictions

Predictions from TargetScan [23,24] were used because we have previously demonstrated the ability of this algorithm to predict known tissue-specific miRNAs [29]. TargetScan's requirement for a perfect match to the seed region and cross-species conservation reduces the false-positive rate [24,25,56]. By minimising background, use of this algorithm maximises the ability to detect effects on expression of real miRNA target genes. Complete miRNA datasets published by Lewis *et al*. [24] were downloaded from the TargetScan website (version 3.1) [57]. For controls, 5 random datasets were generated for each of the gene expression datasets. For each of the random sets, the same numbers of target genes as predicted by TargetScan were randomly assigned to each of the miRNAs. The relationships between candidate miRNAs predicted to affect mRNA levels in the retina and their target genes were analysed using Microsoft Access and 'targets of conserved families' from Targetscan version 4.2. Functional characterisation of predicted target genes was performed using the DAVID bioinformatics database [35].

### Bioinformatic analysis of target mRNA levels

The relative expression of the predicted target gene sets for each miRNA were compared as described previously using stand alone Java and R [58] programs in conjunction with MS Access and MS Excel [29]. Only single gene-specific Affymetrix probesets (suffix_at) were considered and only those expressed in each dataset analysed (designated by a 'present' call if available, or alternatively by a signal strength greater than the median value). Probeset IDs were converted to their cognate Gene Symbols, using the Biomart online suite [59]. For each mRNA expression dataset (Table 4), lists of expressed genes targeted by each miRNA family and their respective expression levels were compiled. The mean expression level was calculated for genes represented by > 1 probeset to provide the 'Average Target Gene Signal'. Only miRNAs with > 50 predicted targets were considered. The expression values of the predicted targets of a specific miRNA in each individual dataset were compared with the set of expression values in that dataset of all the genes predicted to be targeted by miRNAs. To determine whether there was a significant difference between the medians of the ranked gene expression values of the two sets, the nonparametric one-sided Wilcoxon rank sum test was employed. Fisher's combined probability test was used to calculate a composite test statistic from the individual p-values associated with the multiple expression datasets from CE-RSCs, P4 or adult retina[60]. For every miRNA with a combined p < 0.05 we considered that the expression of the set of genes targeted by that miRNA in that tissue was significantly less than the average expression of all the targeted genes.

### RT-PCR

RT for detection of mRNAs was performed with 1 μg of RNA using random hexamers and SuperscriptIII (Invitrogen, Paisley, UK). Specific miRNAs were detected using a modified version of the method described by Shi and Chiang [61], in which mature miRNAs are polyadenylated and target sequences for a reverse primer are subsequently incorporated into cDNA by use of an oligo dT adapter. One microgram of RNA was polyadenylated using poly(A) polymerase (PAP; Ambion, Foster City, CA) at 37°C for 1 hour in a 25-μl reaction mixture. RNAs were then reverse transcribed with 200 U reverse transcriptase (SuperScript III; Invitrogen, Paisley, UK) and 0.5 μg poly (T) adapter (3' rapid amplification of complementary DNA ends (RACE) adapter in the FirstChoice RLM-RACE kit; Ambion). Primers for specific miRNAs were based on miRNA sequences obtained from miRBase [62,63] (Table 5). The reverse primer was the 3' adapter primer (3' RACE outer primer in the FirstChoice RLM-RACE kit; Ambion).

**Table 5:** Sequences of primers and ISH probes.

DNA oligonucleotides	
**Name **	**5'-3' nucleotide sequence**

Let-7d	AGAGGTAGTAGGTTGCATAGT

miR-9	TCTTTGGTTATCTAGCTGTATGA

miR-24	TGGCTCAGTTCAGCAGGAACAG

miR-25	CATTGCACTTGTCTCGGTCTGA

miR-27a	TTCACAGTGGCTAAGTTCCGC

miR-34a	TGGCAGTGTCTTAGCTGGTTGTT

miR-34b	TAGGCAGTGTAATTAGCTGATTG

miR-122	TGGAGTGTGACAATGGTGTTTG

miR-124	TAAGGCACGCGGTGAATGCC

miR-128	TCACAGTGAACCGGTCTCTTTT

miR-125a-5p	TCCCTGAGACCCTTTAACCTGTGA

miR-125b-5p	TCCCTGAGACCCTAACTTGTGA

miR-130a	CAGTGCAATGTTAAAAGGGCAT

miR-130b	CAGTGCAATGATGAAAGGGCAT

miR-150	TCTCCCAACCCTTGTACCAGTG

miR-204	TTCCCTTTGTCATCCTATGCCTG

miR-326	CCTCTGGGCCCTTCCTCCAGT

miR-370	GCCGGCTGGGGTGGAACGTGGTT

miR-378	CTCCTGACTCCAGGTCCTGTGT

miR-485-5p	AGAGGCTGGCCGTGATGAATTC

Poly (T) adapter	GCGAGCACAGAATTAATACGACTCACTATAGGTTTTTTTTTTTTWN

Reverse RACE	GCGAGCACAGAATTAATACGAC

ETS1F	CCAGACAGACACCTTGCAGA

ETS1R	TGAGGCGATCACAACTATCG

KLF13F	GAAGCACAAGTGCCACTACG

KLF13R	GGCAGCTGAACTTCTTCTCG

NFIBF	AGCTGCTGGAAGTCGAACAT

NFIBR	TGAAGGTGGAGGTGGAGTTC

LIN28BF	AAGGCCTTGAGTCAATACGG

LIN28BR	CACTTCTTTGGCTGAGGAGG

SH2B3F	CTGGAGCTCTTCGACCCAC

SH2B3R	ATGTCTGTCCGGTCCTTCAC

ACCN2F	AGCTGTTACCATGGACTCGG

ACCN2R	CACGCAGTACTCCTGGTCCT

	

***in situ *probes**	

**Name **	**Sequence (enhanced with LNA) 5'-3'**

mmu-miR-124	GGCATTCACCGCGTGCCTTA

mmu-miR-34a	AACAACCAGCTAAGACACTGCCA

mmu-miR-128	AAAAGAGACCGGTTCACTGTGA

mmu-miR-125b-5p	TCACAAGTTAGGGTCTCAGGGA

mmu-miR-378	CCTTCTGACTCCAAGTCCAGT

mmu-miR-122	CAAACACCATTGTCACACTCCA

Scrambled-miR	TTCACAATGCGTTATCGGATGT

Oligonucleotide primers and *in situ *probes from the miRCURY™LNA Detection system (Exiqon, Vedbaek, Denmark).

PCR was performed for 45 cycles with denaturation at 94°C for 30 seconds, annealing at 55°C for 30 seconds, and extension at 72°C for 30 seconds (LightCycler 480: Roche, Mannheim, Germany). PCR products were analyzed by polyacrylamide gel electrophoresis (20%; Invitrogen) to confirm the predicted size (approximately 60 bp, including mature miRNA and adapter sequences).

Relative RNA expression was determined by measuring the concentration of template in each sample at the threshold cycle (Ct) from a standard curve (Log concentration against Ct) generated from dilutions of a pooled cDNA sample. Relative expression determined by an alternative method employing delta Ct values and efficiency values calculated from a standard curve gave very similar results (Additional file 2: Figure S1). Starting template copy number was estimated from Ct values using the equation: R_0 _= R_Ct _/(1 + E)^Ct ^where Ct is the threshold cycle, R_Ct _is the fluorescence at this cycle, E is amplification efficiency (calculated from a standard curve) and R_0 _is starting fluorescence, which is proportional to the starting template quantity. Starting template copy numbers estimated using the linear regression efficiency method described by Rutledge *et al *[31] were in broad agreement with the above method (Additional file 2: Figure S1) and mean values from the two methods were calculated.

### *In situ *hybridization (ISH)

Mice were euthanised as described above. Eyes were enucleated, fixed in 4% PFA for 1 h at 4°C and cryoprotected trough series of 10, 20 and 30% sucrose, for 1-3 h, embedded in optimal cutting temperature compound (OCT, Sakura, Japan) and snap frozen in an isopentane bath on dry ice. CE-RSC spheres were collected and fixed in 4% PFA for 30 min at 4°C, cryoprotected in 30% sucrose over night, embedded in OCT and snap frozen on dry ice. Locked nucleic acid probes (miRCURY™ LNA Detection, Exiqon) for the candidate miRNAs and a scrambled miRNA control probe (Table 5), were pre-labelled with digoxygenin (DIG) at the 5' end. 10 μm cryosections were fixed in 4% PFA for 10 minutes, rinsed in 1× PBS and 100 μl of hybridisation mix (50% formamide, 0.3 M NaCl, 20 mM Tris-HCl pH 8, 10% dextran sulphate, 1× Denhardt's solution, 1 mg/ml yeast rRNA) containing 2 μl of probe/ml was applied to sections. Slides were covered with glass cover slips and hybridisation was performed overnight at 20°C below the Tm. Slides were washed twice in 1 × SSC, 50% formamide, 0.1% Tween 20 for 30 min, and once in 1 × SSC, 0.1% Tween 20 and 0.2 × SSC 0.1% Tween 20 for 30 minutes at 65°C. Two washes in 1× MABT (100 mM maleic acid, 150 mM NaCl, 0.1% Tween 20, pH 7.5) were performed at RT for 30 minutes. After incubation in blocking solution containing 1× MABT, 2% blocking reagent (Roche, Mannheim, Germany) and 20% heat inactivated sheep serum (Sigma-Aldrich, Poole, UK) for 2 hours at RT, anti-digoxygenin Fab fragments (Roche, Mannheim, Germany) were applied at 1:5,000 dilution overnight at RT. Detection staining was performed by incubation with the alkaline phosphatase chromogenic substrate 5-bromo-4-chloro-3-indolylphosphate/nitro-blue tetrazolium (BCIP/NBT) (Roche, Mannheim, Germany).

## Authors' contributions

AA performed bioinformatic analyses and some laboratory work; JG-F performed RT-PCR and ISH and contributed to manuscript preparation. LH performed miRNA transfections and mRNA RT-PCR. MD performed microarray analysis with P4 mice; TC contributed to supervision and manuscript preparation; DAS conceived and supervised the study, performed bioinformatic analyses and prepared the manuscript. All authors read and approved the final manuscript.

## Supplementary Material

Additional file 1 **Table S1**. Significance of predicted effects of miRNAs upon mRNA expression in all gene expression datasets (CE-RSCs, P4 and adult retina) and p-values calculated using Fisher's combined probability test. Click here for file

Additional file 2 **Figure S1**. miRNA expression detected by RT-PCR. (A) Comparison of relative miRNA expression calculated using a standard curve (as shown in Figure 3A) with the values calculated using ΔCt and efficiency; for each gene the sample with highest expression was normalized to a value of 100. Each point represents the expression of one miRNA in a single sample as determined by both methods, which yielded very similar results (R^2 ^= 0.983). (B) Starting template copy numbers estimated for each miRNA based on threshold cycle (Ct) and amplification efficiency plotted against the values calculated by a linear regression efficiency method [31](R^2 ^= 0.661). Click here for file

Additional file 3 **Table S2**. All genes predicted to be targeted by candidate miRNAs affecting mRNA levels in each tissue. Genes assessed experimentally for miRNA interactions are highlighted in bold. Click here for file

Additional file 4 **Table S3**. The categories of functional annotations enriched within those genes predicted to be targeted by two or more candidate miRNAs affecting mRNA levels within CE-RSCs, PE or adult retina. Analyses were performed using the DAVID bioinformatics database [35]; categories with p < 0.05 after correction for multiple comparisons (Benjamini) are listed. Click here for file
