# Supplementary figures and images for: Prediction of microRNAs affecting mRNA expression during retinal development
Source: BMC Dev Biol. 2010 Jan 6;10:1. doi: 10.1186/1471-213X-10-1 (PMC2821300; doi:10.1186/1471-213X-10-1)

**A**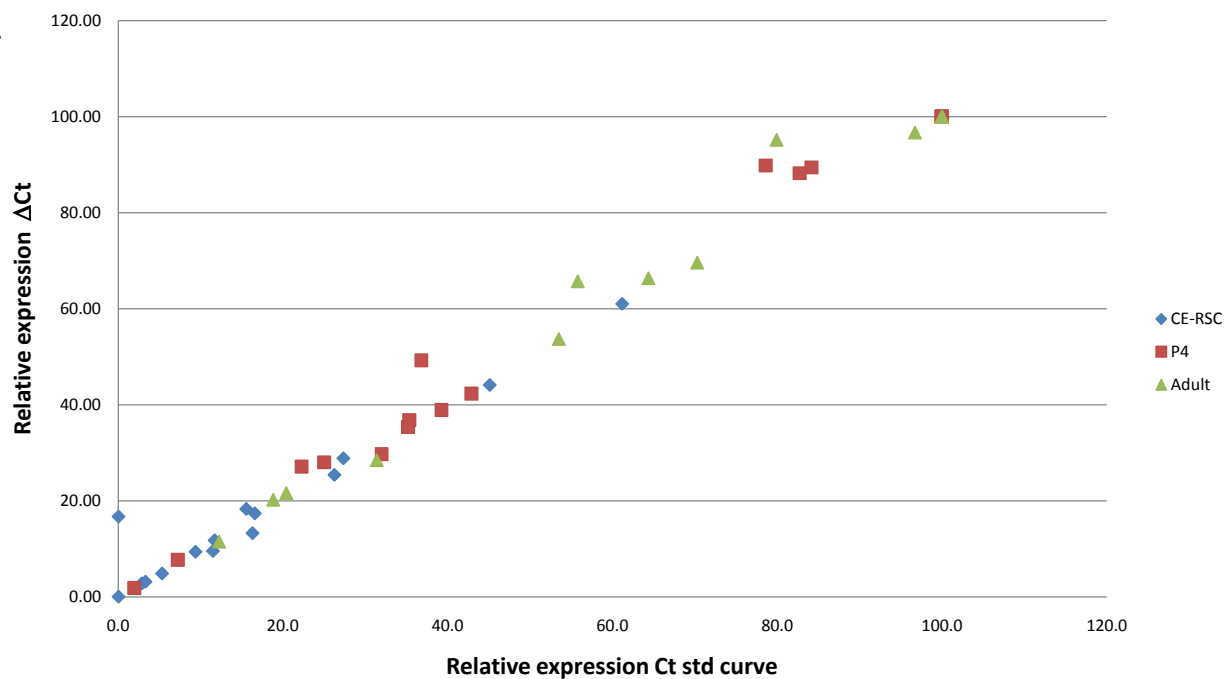

Supplement: Additional file 2 — Figure S1. miRNA expression detected by RT-PCR. (A) Comparison of relative miRNA expression calculated using a standard curve (as shown in Figure 3A) with the values calculated using ΔCt and efficiency; for each gene the sample with highest expression was normalized to a value of 100. Each point represents the expression of one miRNA in a single sample as determined by both methods, which yielded very similar results (R2 = 0.983). (B) Starting template copy numbers estimated for each miRNA based on threshold cycle (Ct) and amplification efficiency plotted against the values calculated by a linear regression efficiency method [31](R2 = 0.661). [file 1471-213X-10-1-S2.PDF]
